# Low-cost Posterior C1–C2 Fusion Using Sublaminar Wiring in Neurologically Intact Young Patient with Type IIA Odontoid Fracture: A Resource-oriented Approach

**DOI:** 10.1055/a-2731-4673

**Published:** 2025-11-12

**Authors:** Carlos Novondo, César Alas-Pineda, Anahi Gisselle Pacheco, Clarisa L. Reyes-Guardado, Kristhel Gaitán-Zambrano

**Affiliations:** 1 Neurocirugía, Hospital Dr. Mario Catarino Rivas, San Pedro Sula, Cortés, Honduras; 2 Neurocirugía, Hospital Militar del Norte, San Pedro Sula, Cortés, Honduras; 3 Quantitative Biomedical Sciences, Geisel School of Medicine, Dartmouth College, Hanover, New Hampshire, United States; 4 Facultad de Medicina y Cirugía, Universidad Católica de Honduras - Campus San Pedro y San Pablo, San Pedro Sula, Cortés, Honduras

**Keywords:** odontoid fracture, C2 fracture, sublaminar wiring, cervical spine trauma, case report, low-cost spinal surgery

## Abstract

**Purpose:**

To present a low-cost posterior cervical fixation technique using sublaminar wiring for the management of a comminuted odontoid fracture (type IIA) in a young, neurologically intact patient, emphasizing its relevance in low-resource environments where advanced instrumentation is inaccessible.

**Methods:**

A 21-year-old male sustained a comminuted fracture of the odontoid process of C2 following a high-impact motorcycle accident. Upon admission, he was alert, fully oriented, and neurologically intact. Imaging revealed a comminuted type IIA odontoid fracture without displacement. Due to the patient's economic limitations and the unsuitability of the fracture for anterior fixation, a posterior approach using double sublaminar wiring between C1 and C2 with autologous iliac crest bone graft was performed.

**Results:**

The procedure was successfully completed with no intraoperative or postoperative complications. The patient was discharged on postoperative day 2 and demonstrated excellent recovery. Follow-up imaging at 3 months confirmed over 90% fracture consolidation. Imaging at 6 months was requested but not obtained due to loss of follow-up. Functional recovery was complete, with only a partial reduction in cervical range of motion as expected with C1–C2 fusion.

**Conclusion:**

Sublaminar wiring offers a safe, effective, and affordable alternative for posterior fixation of comminuted odontoid fractures, particularly in young patients and in settings where access to advanced spinal instrumentation is limited.

## Introduction

The odontoid process of the second cervical vertebra is a bony projection arising from the vertebral body of C2. It functions as a fulcrum for the lateral rotation of the ring of C1 or atlas over the body of the axis. 1 Traumatic axis injury is a common cervical spine injury, accounting for more than 20% of cervical fractures. The odontoid process of C2 is the main site of axis injury. Odontoid fracture accounts for 5 to 18% of C2 injuries and 10 to 19% of all fractures in the cervical region. 2 Multiple axis fractures account for only 1% of cervical spine fractures. 3

The etiology of odontoid fractures varies according to the patient's age; in young people, the main cause is high impact, such as traffic accidents, sports injuries, or falls from great heights. In older people, it is the result of low-impact trauma that usually occurs when slipping and falling on the same plane of support 4 5 and pathologies that compromise bone density. 6

Hyperflexion of the spine is the most frequent mechanism of injury; it induces anterior displacement of the odontoid process of the axis. Hyperextension causes posterior displacement. 7 Lateral flexion movements, compression, or rotational forces are also mechanisms that cause odontoid fractures. 8 9 Symptoms are not specific, the most common being severe neck pain, limited cervical spine mobility, and headaches. Some patients experience life-threatening neurological complications following the injury, such as spinal shock, severe neurological deficits, and even respiratory disorders. 10

There are no standardized treatment guidelines for odontoid fractures. Therapeutic options should be individualized to the patient's characteristics, experience, and the surgeon's available resources. Conservative treatment includes external immobilization and use of devices including rigid collar or halo vest immobilization. 5 11 Surgical treatment includes anterior fixation/posterior fixation. However, the most efficient treatment strategy is still under debate. 12 Several factors negatively affect bone healing such as nicotine, alcohol, the force of impact, mechanism of injury, bone quality, age, and a history of cervical spine surgeries are also of significance. 10

The trauma in the odontoid process of C2 was treated conservatively until 1910 when posterior fixation with wires began to be used as a surgical treatment, a procedure that has evolved since Mixter and Osgood first described it. 9 13

This case report aims to describe a low-cost surgical technique and its favorable evolution in the treatment of a young patient with multiple odontoid fractures of the axis, a case in which early age of presentation and trauma mechanism are typically associated with increased mortality. After surgery, the patient had no sequelae, with a return to work and full recovery of quality of life.

## Illustrative Case

A 21-year-old male patient, from an urban area, with no prior pathological, psycho-social, or past intervention history of interest, suffered high-impact trauma in a motorcycle rollover road accident. He was transferred while unconscious to the emergency department of a private hospital for stabilization. He was later referred to the Hospital Militar del Norte in San Pedro Sula, Honduras, for further specialized treatment.

**Fig. 1:**
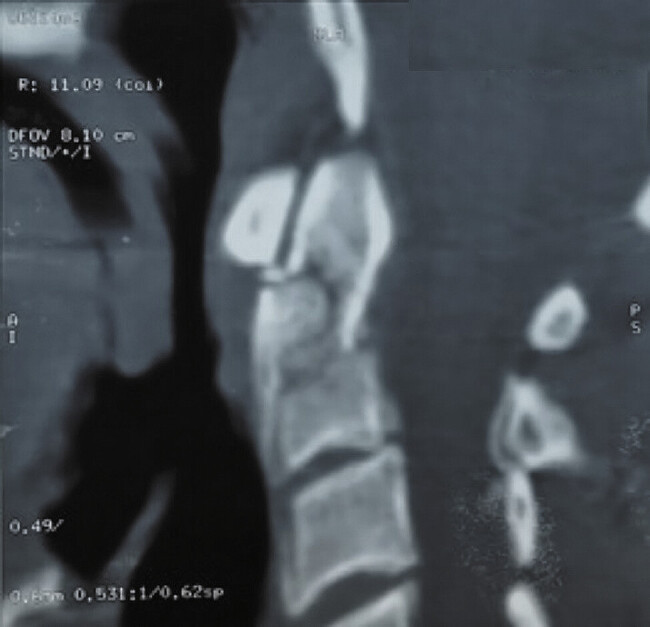
Cervical computed tomography with simple reconstruction. Comminuted fracture of the odontoid process of the second cervical vertebra.

He was admitted to the emergency room wearing a rigid Philadelphia-type cervical collar and the initial evaluation revealed a Glasgow Coma Scale score of 15, no respiratory distress, and isochoric pupils. Intentional neurological examination showed a motor strength of 5/5 on the Daniels scale in all four extremities and non-referred neurosensory alterations. Ecchymosis on the head and multiple abrasions on the body were present. On admission, the patient reported moderate to severe pain in the posterior craniocervical junction, with significant limitations to the lateral rotation of the head and severe cervical muscle spasms. There were no signs of spinal cord compression. Blood biometry and blood chemistry studies were within normal parameters. Cerebral tomography revealed no abnormalities in encephalic structures, and cervical tomography evidenced comminuted odontoid fracture of C2 without apparent displacement in the cervical region ( Fig. 1 ).

On day 4 of hospitalization, a posterior surgical approach was performed due to the comminution of the odontoid process of C2, a determining factor that contraindicated an anterior approach with an odontoid screw. Due to the patient's economic limitations and lack of medical insurance to cover the costs of a posterior transpedicular screw system and lateral mass screws, we opted for sublaminar wiring.

**Fig. 2:**
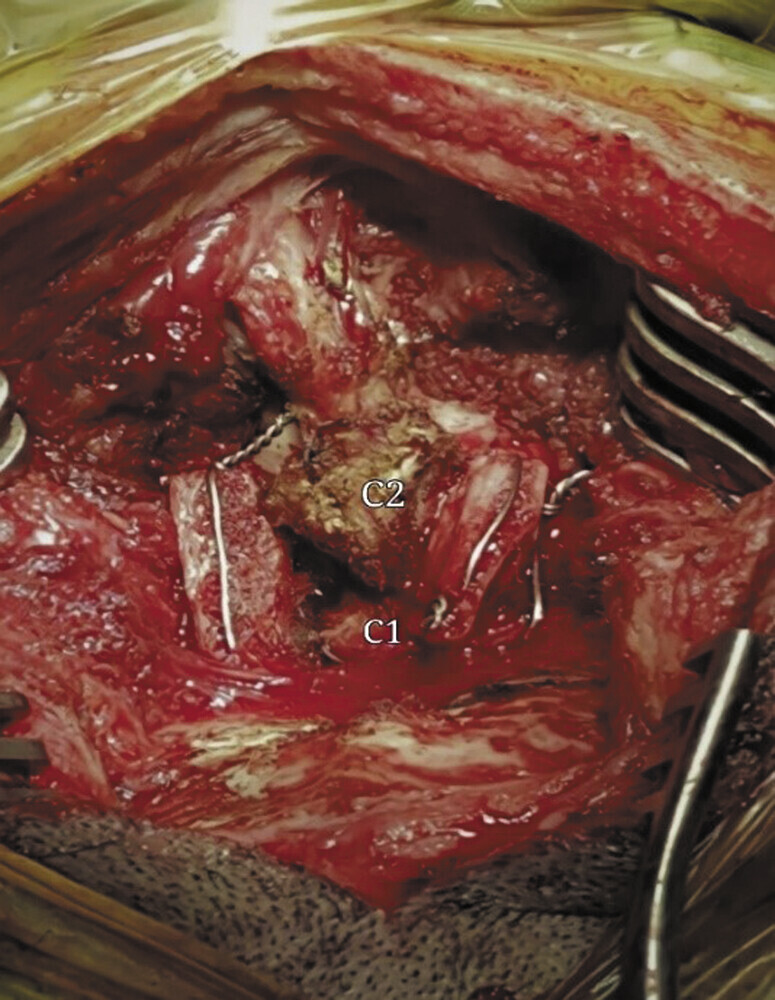
Intraoperative photograph showing simple sublaminar wiring with bilateral iliac crest bone graft.

**Fig. 3:**
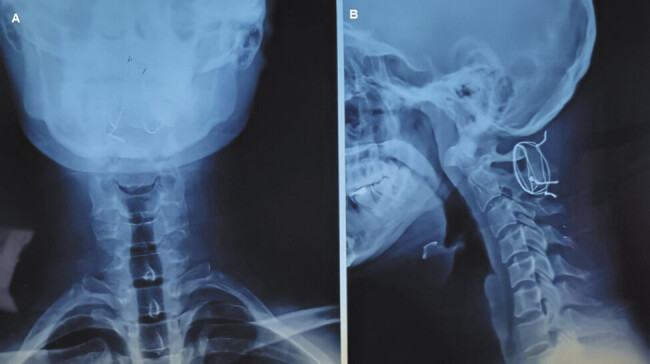
Postoperative cervical X-ray: ( **A** ) anteroposterior projection and ( **B** ) lateral projection.

The patient was taken to the operating room, where general anesthesia was administered following asleep intubation. The patient was positioned prone with the head in a neutral alignment. Prophylactic antibiotic therapy with cefazolin 1 g IV STAT was administered 30 minutes prior to the surgical incision. A midline incision and subperiosteal dissection were performed using electrocautery and elevators to expose the occipital bone, C1, C2, and C3. An autologous bone graft was first harvested from the patient's left iliac crest. Following decortication of the posterior arch of C1 and the laminae of C2, double sublaminar wiring was performed bilaterally between C1 and C2 using 0.7-mm orthopedic cerclage wire passed from inferior to superior. Once satisfactory reduction was achieved, bilateral simple wiring was completed, followed by secure placement of the autologous bone graft over the decorticated area. The procedure lasted approximately 2.5 hours, with an estimated blood loss of 120 mL. No intraoperative complications were encountered during the procedure ( Figs. 2 and 3 ).

The patient left the operating room wearing a Philadelphia collar, extubated, with no motor deficit. He was transferred to the intermediate intensive care unit for monitoring. He was prescribed antibiotic coverage for 2 days, painkillers post-operation, and discharged on postoperative day 2.

**Fig. 4:**
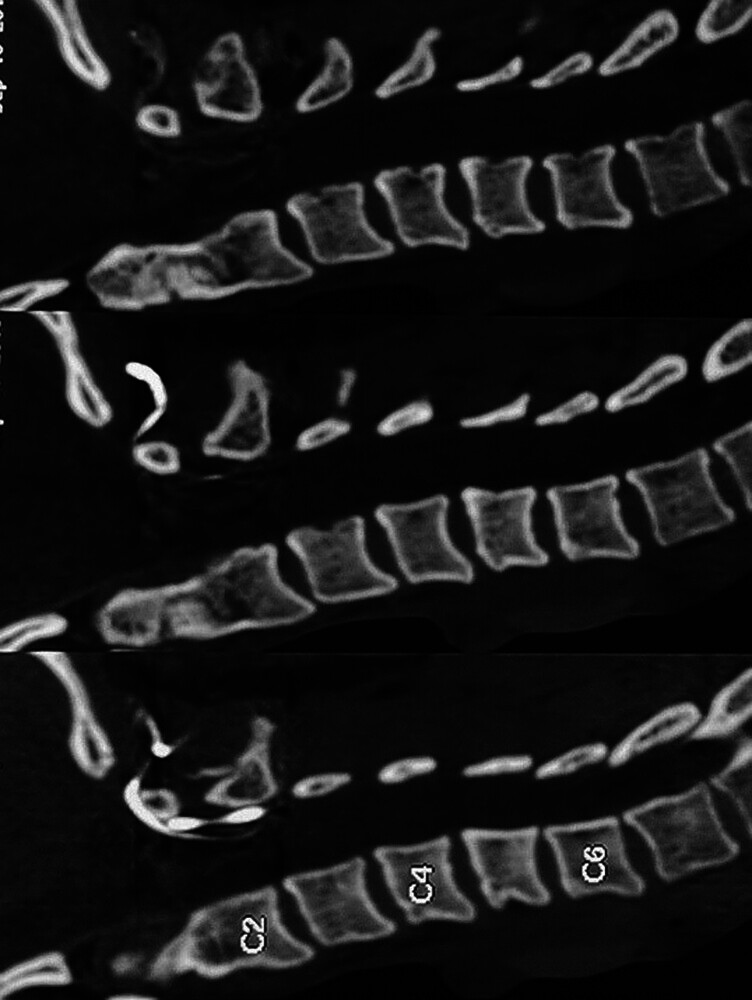
Cervical tomographic control 3 months after the procedure: simple reconstruction in sagittal section showing osteosynthesis material and resolution of more than 90% of the fracture.

The post-surgery treatment of this patient included the use of a Philadelphia collar, which was indicated for 3 months after the procedure; at the end of this period the patient was told that he was free to move his neck if the CT scan demonstrated that the fusion was successful. However, the patient only used it for 1 week, but there were no complications during recovery. In functional terms, the procedure and recovery were deemed successful, despite an approximately 50% reduction in cervical range of motion, which is within the expected range in C1–C2 fusion. Additionally, two control appointments were made with cervical tomography at 3 and 6 months post-operation. Although the 6-month postoperative imaging and follow-up appointment were requested, the final CT scan could not be obtained due to loss of patient follow-up. However, clinical recovery was complete by 3 months, with functional return to work and no reported complications. The 3-month CT scan demonstrated more than 90% resolution of the fracture and stable osteosynthesis. At the time, the patient was asymptomatic and maintained a preserved quality of life ( Fig. 4 ). In other cases, the long-term follow-ups consist of CT scans a year after the procedure and standard X-rays to study fusion every second year for the average 7-year follow-up which in our case was not possible due to loss of patient follow-up.

## Discussion

Fracture of the second cervical vertebra (C2) is the most common cervical spine injury in the geriatric population, accounting for 69% of cases with 36% in young adults. 4 5 The neurological risk associated with odontoid fracture of C2 is potentially fatal, due to the proximity to the spinal cord, and the mobility of the atlantoaxial joint, which greatly increases the risk of instability. For these reasons, the associated morbidity and mortality is high. 2 14 Deficits occur in 8.5% of cases, and 2.4% of mortality is due to neurological deficits. 5

This patient suffered a comminuted fracture at a cervical level in a high-impact road accident while riding a motorcycle, with transient loss of consciousness and no neurological deficit, and survived for subsequent stabilization and transfer without motor impairment. According to the recommendations of Ryken et al, for the initial management of non-displaced odontoid fractures type I, type II, and type III, external cervical immobilization is recommended. 15

In contrast, type II and III fractures with displacement ≥5 mm and comminution of the odontoid require surgical intervention indicated for unstable odontoid fractures associated with neurological risk and high non-union ranges 8 for stabilization and fusion of the lesion. 15

There are multiple options for the fusion of the C1–C2 joint, including screws to the lateral masses of C1, transpedicular screws to C2, and translaminar screws and posterior wires. The primary goal in performing any of the procedures is to align and stabilize the upper cervical spine. 16

In our case, a posterior approach using sublaminar wiring was chosen due to the comminuted nature of the fracture and the lack of equipment in addition to the patient's inability to afford more advanced instrumentation. The posterior technique requires intact posterior elements and allows for bone grafting with acceptable fusion rates. The Gallie technique, one of the most widely used sublaminar wiring methods, involves placing a bone graft and securing it with sublaminar wire below the spinous process of C2 and around the arch of C1. 5 17 We used a variation of this technique, placing the graft over the C1 arch and using bilateral double sublaminar wiring.

If advanced instrumentation had been accessible and economically feasible for the patient, posterior screw fixation would have been considered. However, sublaminar wiring was selected as a viable and ethical alternative in this context.

Although posterior wiring techniques have existed for over a century, recent comparative studies provide valuable context. In a 2018 study comparing modified Gallie fusion with posterior cervical screw constructs for type II odontoid fractures, both groups achieved similar fusion rates of >88% and functional outcomes. However, the wiring group demonstrated shorter operative time, lower blood loss, and significantly reduced procedural costs. 18 Despite slightly lower patient satisfaction scores, the technique remains viable in resource-limited settings.

Biomechanically, screw–rod constructs offer superior rotational stability, but these are costlier and more technically demanding. The screw implantation is a high-risk procedure, as VA injury or spinal cord injury may occur due to malpositioned screw. Although clearly described in the literature the anatomical identification does not always work, because actual intraoperative entry point and trajectory of the screws may alter due to the varied surgical position, surgeons' experience, fracture displacement, and intraoperative weight traction. 18

Posterior wiring remains appropriate for young patients with good bone quality and reducible fractures, particularly in resource-limited settings where advanced implants are unavailable. 18 Although posterior wiring has disadvantages including risks of wire migration, reduced stability in osteoporotic patients, and permanent reduction in C1–C2 motion, these are acceptable trade-offs in select patients. Due to these disadvantages this technique is not appropriate to implement in the geriatric population, where bone mass loss is significant, and canal narrowing is predominant due to aging.

The procedure provides strong bony fixation, with a high healing rate. However, the sacrifice of atlantoaxial rotational function is unavoidable. It is a viable option when the anterior approach is contraindicated in comminuted fracture, unfavorable anteroposterior fracture plane angulation, or rupture of the transverse ligament of C1–C2. 19

Among the disadvantages of the posterior wiring technique, it is important to note that these are related to the fact that these can only be applied safely when posterior elements are intact, and the bone arches are adequate. A risk of spinal cord injury can be associated with the passage of the bands below the C1 arch. 20

In addition, the C1–C2 complex allows 50% of cervical spinal rotation, 10 degrees of flexion–extension and 50 degrees axial rotation. Therefore, the fusion of these segments will undoubtedly cause permanent loss of this movement. The anatomy and location of this complex along with the transfer of kinetic energy contribute to a wide variety of fractures. 12 21

Our technique of choice was the posterior approach, which involves the loss of lateral rotation of the atlantoaxial joint. The patient was informed of the potential sequelae and agreed to undergo the surgical procedure, judging that the benefits outweigh the surgical risks. The condition of the ligaments is the most important factor in the selection of treatment. The main elements that guide surgical treatment are the involvement of the ligamentous complex at the occipital–cervical junction, in the atlantoaxial complex, and in the complex between the second and third cervical vertebrae. 22 Despite the integrity of the articular ligamentous complex, we decided to intervene surgically due to the instability and potential neurological risk caused by the odontoid fracture. Given the equipment limitations and the patient's economic constraints, this was deemed the most appropriate option.

This case supports the selective use of posterior wiring as a context-sensitive solution where high-cost fixation systems are impractical and highlights the need for adaptable surgical strategies in global spine care.

## Conclusion

Despite the challenges experienced in managing our patient, this case highlights how the posterior approach is a viable option when the anterior approach is not possible due to the comminuted nature of the fracture and associated complications, even though it involves some degree of compromise in the rotation of the C1–C2 joint. This approach underscores the importance of carefully weighing risk–benefit factors, particularly patients with limited financial resources and in regions with restricted access to advanced medical technologies and equipment. It offers a low-cost option for maintaining a preserved quality of life to our patients.
